# Meningovascular Neurosyphilis Presenting as Multiple Ischemic Infarcts in a Young Adult

**DOI:** 10.7759/cureus.36405

**Published:** 2023-03-20

**Authors:** Christopher J King, Teresa H Ngo, Martin Constante

**Affiliations:** 1 Neurology, Western University of Health Sciences, Pomona, USA; 2 Neurology, San Antonio Regional Hospital, Upland, USA

**Keywords:** acquired immune deficiency syndrome (aids), infectious aortitis, treponema pallidum, ischemic infarct, stroke, immunosuppression, hiv, meningovascular neurosyphilis, neurosyphilis, syphilis

## Abstract

Neurosyphilis occurs when the spirochete *Treponema pallidum* invades the cerebrospinal fluid (CSF). Clinical presentation depends on an individual's immune response and invasion location, with all possible involvement of meningeal, vascular, and/or parenchymatous tissues. Meningovascular neurosyphilis occurs when both the meninges and vasculature are affected and can lead to headaches, photophobia, neck stiffness, cranial nerve palsies, and/or ischemic brain infarctions due to infectious arteritis. The following report describes the rare case of a 32-year-old male patient presenting with multiple ischemic brain infarctions of varying ages. The stepwise diagnostic approach as described allowed the medical team to reach the final diagnosis of meningovascular neurosyphilis with concomitant acquired immunodeficiency syndrome (AIDS). This case emphasizes the importance of maintaining high clinical suspicion in all young adult patients who present with acute neurological deficits.

## Introduction

Neurosyphilis refers to infection of the central nervous system by the spirochete *Treponema pallidum*. Historically, neurosyphilis was often described as a form of “tertiary syphilis,” but it is now established that neurosyphilis can occur at any stage of infection (primary, secondary, latent, or tertiary) [1]. Invasion of the cerebrospinal fluid (CSF) with *T. pallidum* can result in spontaneous resolution without an inflammatory response in some cases, while in others, persistent meningitis will develop due to failure to clear organisms from the CSF [2]. Clinically, neurosyphilis can manifest itself with meningeal, vascular, or parenchymatous symptoms [3]. Meningeal symptoms include headache, photophobia, neck stiffness, and cranial nerve palsies [3]. Vascular involvement manifests as infectious arteritis that can affect any vessel from the aorta to those within the subarachnoid space surrounding the brain or spinal cord and could result in infarction [1,2]. Parenchymal neurosyphilis presents with neurodegenerative symptoms, including memory deficits, emotional lability, psychosis, and/or tabes dorsalis [4,5]. Meningeal and vascular involvement frequently coexist (meningovascular neurosyphilis) and commonly present as an ischemic stroke in a young adult anywhere from the first months to years after the initial untreated infection [1,2].

In the pre-antibiotic era, neurosyphilis occurred in 25-30% of patients with syphilis, with meningovascular syphilis comprising only 10% of cases [2,6]. In the Los Angeles area today, the occurrence of neurosyphilis is closer to 1.5%, with a large percentage occurring in the setting of human immunodeficiency virus (HIV) infection [7,8]. Therefore, clinical suspicion for co-infection should be elevated for all patients with manifestations of either neurosyphilis or HIV. The following case demonstrates this relationship and provides an example of the diagnostic process of neurosyphilis in a young adult patient.

## Case presentation

A 32-year-old male presented to the emergency department one day after reportedly feeling “confused” while at work, where he later began experiencing tingling paresthesia and weakness affecting his right upper and lower extremities. After subsequently returning home, the patient was found to be confused and somnolent, answering questions with occasional inappropriate words, so was brought to the hospital. Upon questioning, the patient reported intermittent headaches, palpitations, and almost daily diarrhea for the last month. Additionally, the patient voluntarily conveyed a history of methamphetamine and marijuana use, along with recent unprotected sexual practices. Vital signs on admission were within normal limits. Pertinent neurological examination displayed minimally reactive pupils to light bilaterally, pupillary asymmetry (the left pupil measured 3 mm and the right pupil measured 5 mm), and no residual weakness or neurological deficits. Seborrheic dermatitis was also noted.

**Figure 1:**
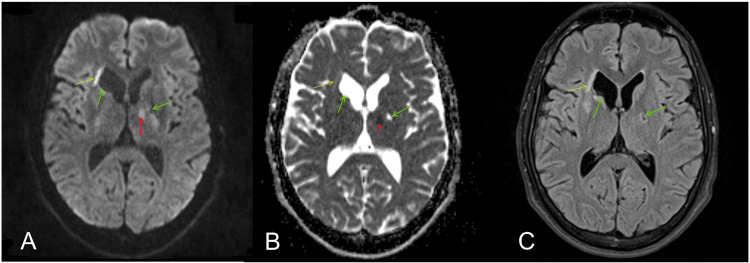
MRI brain scan axial plane sequences DWI (A), ADC (B), and T2-weighted FLAIR (C) showing restricted diffusion of an acute infarction at the left anterior thalamus (red arrows), subacute infarction at the periventricular region of the anterior horn of the right lateral ventricle (yellow arrows), and lesions at the left anterior lateral thalamus and right head of the caudate nucleus that represents the gliosis of chronic infarctions (green arrows). MRI: magnetic resonance imaging; DWI: diffusion-weighted imaging; ADC: apparent diffusion coefficient; FLAIR: fluid-attenuated inversion recovery

**Figure 2:**
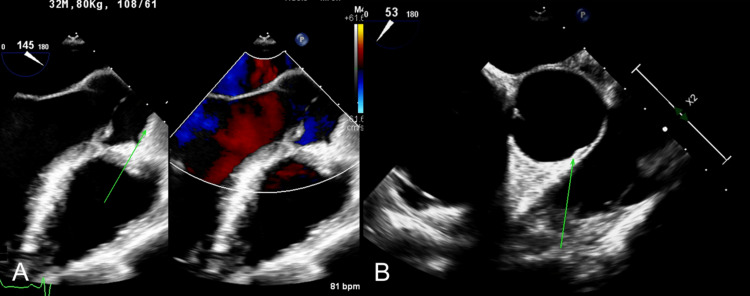
TEE mid-esophageal long axis view with and without Doppler (A) and ascending aorta short axis view (B) showing mild thickening of the ascending aortic wall (green arrow), suggestive of aortitis. TEE: transesophageal echocardiogram

Computed tomography angiography (CTA) of the carotid and vertebral arterial systems displayed no evidence suggestive of acute large artery thrombosis. Continuous cardiac telemetry showed no evidence of atrial fibrillation or arrhythmias. Magnetic resonance imaging (MRI) of the brain showed acute, subacute, and chronic infarctions in multiple different vascular territories (Figure 1). A transthoracic echocardiogram (TTE) showed a possible hyperechoic structure in the left atrium that was poorly visualized. A follow-up transesophageal echocardiogram (TEE) with bubble study showed no intracardiac thrombus and no interatrial septum shunting. The TEE did, however, demonstrate mild thickening of the ascending aorta suggestive of aortitis (Figure 2). Venous ultrasound of the lower extremities revealed no evidence of deep vein thrombosis.

The patient's pertinent serum and CSF laboratory findings are summarized in Table 1. Initial laboratory values showed normocytic anemia with low hemoglobin, low hematocrit, and normal mean corpuscular volume. The erythrocyte sedimentation rate was elevated, and the activated partial thromboplastin time was normal. The serum rapid plasma reagin (RPR) test was positive which triggered further diagnostic investigation. A lumbar puncture was performed on day three. Findings in the CSF included elevated red blood cells (RBCs), elevated white blood cells (WBCs) with neutrophilic predominance, low glucose (serum glucose was within normal limits), and elevated protein. These findings were consistent with meningoencephalitis. On day four, the cryptococcal antigen latex agglutination test returned negative. On day five, the HIV-1 antibody test returned positive along with an elevated quantitative polymerase chain reaction test. Additionally, there was a significant reduction seen in the patient's CD4 count, indicating immunosuppression due to acquired immunodeficiency syndrome (AIDS). With the combination of meningoencephalitis and aortitis shown on TEE, the patient was started on intravenous penicillin G as per protocol. Serum *T. pallidum* micro-hemagglutination assay and CSF venereal disease research laboratory (VDRL) tests later returned positive, confirming the diagnosis of meningovascular neurosyphilis.

**Table 1:** Pertinent serum and CSF laboratory examination values. CSF: cerebrospinal fluid; MCV: mean corpuscular volume; ESR: erythrocyte sedimentation rate; aPTT: activated partial thromboplastin time; RPR: rapid plasma reagin; MHA-TP: *Treponema pallidum* micro-hemagglutination assay; HIV-1 qPCR: human immunodeficiency virus-1 quantitative polymerase chain reaction; RBC: red blood cells; WBC: white blood cells; VDRL: venereal disease research laboratory

Examination		Patient result	Reference range
Serum	Hemoglobin	10.9 g/dL	14-18 g/dL (male)
	Hematocrit	34%	42-50% (male)
	MCV	81 fL	80-98 fL
	ESR	89 mm/h	0-15 mm/h
	aPTT	32.7 seconds	25-35 seconds
	RPR	Reactive	-
	MHA-TP	Reactive	-
	Glucose	111 mg/dL	<140 mg/dL (non-fasting)
	HIV-1 antibody	Reactive	-
	HIV-1 qPCR	133,000 copies/mL	Not detected
	CD4 T-lymphocytes	76 cells/mm^3^	500-1,500 cells/mm^3^
CSF	RBC	4 cells/µL	0 cells/µL
	WBC	210 cells/µL	cells/µL
	1. Neutrophils	64%	0-6%
	2. Lymphocytes	25%	40-80%
	3. Monocytes	11%	15-45%
	Glucose	26 mg/dL	2/3rds serum glucose
	Protein	200.3 mg/dL	<45 mg/dL
	Cryptococcal antigen latex agglutination test	Negative	-
	VDRL	Reactive	-

## Discussion

The presentation of acute right-sided weakness, numbness, and tingling paresthesia along with minimally reactive and asymmetric pupils to light was highly suggestive of acute brain infarction. The patient's altered mental status favored either a thalamic involvement or a more diffuse encephalopathic pattern, possibly associated with infection. MRI of the brain displayed an acute infarct of the left anterior thalamus along with multiple other infarcts of varying ages and in different vascular territories (Figure 1). Adults aged 18-45 years make up only 10-14% of ischemic strokes, and their etiology displays greater heterogeneity than in older individuals [9]. Due to this patient’s history of recent methamphetamine use, along with confirmatory urine toxicology, vasoactive substance use as a causative agent had to be considered. In recent literature, methamphetamine and other vasoactive substances have been associated with strokes in young adults, though most cases displayed a hemorrhagic rather than ischemic nature of infarction [10]. With this knowledge, methamphetamine use as the sole causative agent for our patients’ multiple ischemic infarcts was deemed improbable therefore other inflammatory and infectious causes were thoroughly explored [9].

Given our patient's positive RPR test along with key clinical findings within the history and physical examination - daily diarrhea for approximately one month and the detection of seborrheic dermatitis - suspicion was seriously raised for severe immunosuppression. Over the last 20 years, the number of primary and secondary syphilis cases has been increasing in the United States and Europe [11]. Neurosyphilis in the modern era is mostly found in persons with HIV co-infection [8,11]. Seborrheic dermatitis alone occurs in 85-95% of patients with advanced HIV infection, while gastrointestinal complaints, especially diarrhea, are one of the most common [12,13]. Given the high likelihood of co-infection with syphilis and HIV, a lumbar puncture was warranted at this time. Investigations were then further narrowed to infectious diseases, other than syphilis itself, known to cause ischemic strokes, specifically in those with concomitant immunosuppression from HIV infection [11].

Bacterial meningitis can lead to ischemic strokes in 15-25% of cases [14]. CSF makeup typically includes a WBC count of 1,000-5,000 cells/mm^3^ with a neutrophil predominance of 80-95%, a glucose concentration <40 mg/dL with a CSF to serum glucose ratio of ≤0.4, and a protein concentration >220 mg/dL [15]. This patient's CSF values did not conclusively match that of untreated bacterial meningitis therefore, we relied on the results of a Gram stain, which demonstrates a specificity of ≥97%, and a CSF culture, which is positive in 70-85% of patients [15]. Both tests returned as negative, ruling out bacterial meningitis in this patient.

Cryptococcus species are yeasts that can invade the central nervous system leading to meningitis. In the developed world, approximately 50% of cases are associated with HIV infection [11]. Cryptococcal meningitis has been associated with lacunar cerebrovascular infarctions of varying ages, numbers, and locations with the basal nuclei being most affected [16]. In this patient, multiple infarcts affecting the basal nuclei raised serious suspicion for cryptococcal meningoencephalitis. CSF characteristically displays a WBC count of <50 cells/µL (with a mononuclear predominance), a slightly elevated protein level, and a low or normal glucose concentration [17,18]. Our patient’s CSF did not match these characteristic findings, and the highly specific cryptococcal antigen latex agglutination test of the CSF fluid came back negative, ruling out Cryptococcus spp. infection.

A key finding on the transesophageal echocardiogram was aortitis of the ascending aorta. There are many possible causes of aortitis including rheumatological conditions, infectious diseases, or isolated idiopathic cases [19]. Given the concomitant clinical and laboratory evidence along with a negative rheumatological laboratory workup, syphilitic infection had to be highly suspected as the causative agent of this patient's inflammatory response. Syphilitic aortitis is a rare finding and has a strong association with aortic aneurysm formation, therefore treatment was initiated as soon as this diagnosis was suspected [19]. Penicillin G is the treatment of choice for all stages of syphilis infection, including those with concurrent HIV co-infection, with early initiation often leading to a good prognosis [20].

## Conclusions

This 32-year-old male patient displayed multiple ischemic brain infarctions of varying ages. The thorough diagnostic approach, as described, allowed the medical team to consider and rule out multiple possible causes of this patient's presentation, leading to the final diagnosis of meningovascular neurosyphilis with concomitant acquired immunodeficiency syndrome (AIDS). After treatment with intravenous penicillin G, the patient displayed clinical improvement during his hospital stay. He received extensive counseling, case-management guidance, and infectious disease involvement to initiate long-term management and follow-up. This case exemplifies the importance of considering the diagnostic workup for any young adult patient with acute neurological deficits, including exploring immunosuppression via HIV and other infectious causes, specifically meningovascular neurosyphilis.
